# Biomarker for Cancer: A great Promise for Future

**DOI:** 10.4021/wjon352w

**Published:** 2011-08-24

**Authors:** Dugeshwar Karley, Deepesh Gupta, Archana Tiwari

**Affiliations:** a School of biotechnology, Rajiv Gandhi Proudyogiki Vishwavidyalaya Bhopal, M.P. India

**Keywords:** Biomarker, Cancer, Oncoproteomics

## Abstract

Cancer is an uncontrolled growth of a cell due to failure in the cell growth signaling system. Cure of cancer is done only by the complete removal of cancerous cells from the body, the process may sound simple but its implementation is almost impossible. There are number of problems regarding its treatment such as its early detection. Science is progressing every second and so are our expectations. This review is about present scenario and future promises about the development of biomarkers. It is a big challenge whose completion will be beneficial in the early detection of cancer. As early detection is half victory in any disease, especially cancer, this kind of research will be advantageous in the field of cancer treatment.

## Introduction

Cancers represent group of unprecedentedly heterogeneous diseases that affect humans with high frequency and contribute in significant manner to overall morbidity and mortality [1]. usually, an accumulation of DNA alterations irreversibly transforms normal cell into dysplastic and cancerous one. Molecular alterations are frequently found in processes mediated by growth factors, hormones and cytokines via their receptors. Alterations of many other genes directly or indirectly involved in the carcinogenesis and tumor progression process have been demonstrated. Each year, the American Cancer Society estimates the number of new cancer cases and deaths expected in the United States in the current year and compiles the most recent data regarding cancer incidence, mortality, and survival based on incidence data from the National Cancer Institute, the Centers for Disease Control and Prevention, and the North American Association of Central Cancer Registries and mortality data from the National Center for Health Statistics. Incidence and death rates are age-standardized to the 2000 US standard million population. A total of 1 529 560 new cancer cases and 569 490 deaths from cancer occurred in the United States in 2010. Overall cancer incidence rates decreased in the most recent time period in both men (1.3% per year from 2000 to 2006) and women (0.5% per year from 1998 to 2006), largely due to decreases in the 3 major cancer sites in men (lung, prostate, and colon and rectum) and two major cancer sites in women (breast and colorectum) [2].

**Figure 1:**
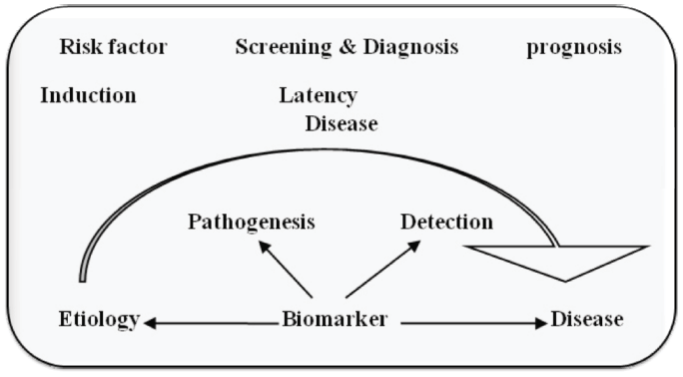
Working of biomarkers.

Most of currently used conventional anti-cancer treatments include surgery, radiotherapy and chemotherapy. Previous to treatment for cancer we need to detect its presence at any body part specifically, and to do so we need an agent i.e. a biological marker or biomarker. According to the definition developed by the NIH, a biomarker is “a characteristic that is objectively measured and evaluated as an indicator of normal biologic processes, pathogenic processes, or pharmacologic responses to a therapeutic intervention”. Biomarkers may be for example certain proteins present on the tumor or released by the tumor in the blood, which indicate the recurrence of the disease after a curative surgical intervention, a single nucleotide polymorphism (SNP) haplotype correlated to the risk that patients will develop a certain drug-related toxicity, the expression level of mRNA or the presence of a gene mutation targeted by a drug, but even the metabolic activity of the tumor, measured by the standard uptake value (SUV) of images obtained during positron emission tomography (PET) examination, or the number of circulating tumor cells may as well considered biomarkers. By another definition biological markers (biomarkers) have been defined as “cellular, biochemical or molecular alterations that are measurable in biological media such as human tissues, cells, or fluids [3]. More recently, the definition has been broadened to include biological characteristics that can be objectively measured and evaluated as an indicator of normal biological processes, pathogenic processes, or pharmacological responses to a therapeutic intervention. In practice, biomarkers include tools and technologies that can aid in understanding the prediction, cause, diagnosis, progression, regression, or outcome of treatment of disease, for example the nervous system there is a wide range of techniques used to gain information about the brain in both the healthy and diseased state, these may involve measurements directly on biological media (e.g., blood or cerebrospinal fluids) or measurements such as brain imaging which do not involve direct sampling of biological media but measure changes in the composition or function of the nervous system. Working of biomarkers can be understood by Figure 1. This shows that in every disease there is a latency period and in this stage if we are able to detect the disease we can go for treatment before it become lethal. Biomarker detects it earlier and helps us to provide treatment for disease as in case of cancer it is more important to detect it earlier, if we can’t detect, it becomes incurable in later stages. Therefore biomarker is extremely important for early detection and treatment of cancer.

## Properties of Biomarkers

The ideal biomarker mark out the events between exposure and disease, perform establishment of dose response, identifies early events in the natural history, identifies mechanisms by which exposure and disease are related, reduces mis-classification of exposures or risk factors and disease, established variability and effect modification, enhanced individual and group risk assessments. Desirable characteristics of biomarker for cancer are shown in Table 1 [4]. In medicine; a biomarker is a term often used to refer to a protein measured in blood whose concentration reflects the severity or presence of some disease state. More generally a biomarker is anything that can be used as an indicator of a particular disease state or some other physiological state of an organism. A biomarker can be a substance that is introduced into an organism as a means to examine organ function or other aspects of health. For example, rubidium chloride is used as a radioactive isotope to evaluate perfusion of heart muscle. It can also be a substance whose detection indicates a particular disease state, for example, the presence of an antibody may indicate an infection [5]. There are some proteins which play role in signaling process, as their concentration level may show reflection during uneven function of any organ or constituents of body. It indicates a change in expression or state of a protein that correlates with the risk or progression of a disease, or with the susceptibility of the disease to a given treatment. Biomarkers are characterized biological properties that can be detected and measured in parts of the body like the blood or tissue. According to Food and Drug Administration (FDA) an ideal biomarker should be specific, sensitive, predictive, robust, simple, accurate, and inexpensive. It should be used in standard biological sources such as serum and urine as the basis of measurement [6].

**Table 1:** Desirable Characteristics of Biomarker for Cancer

Purpose	Non-invasive	Low cost	Simple to perform	Accurate	Informative (discriminatory)
Screening	+++	+++	+++	+++	+++
Predisposition	+++	+++	+++	+++	+++
Early detection	++	++	++	+++	+++
Prognosis	+	+	+	++	++
Drug response	+++	++	++	+++	+++
Target for drug	NA	+	NA	+++	NA

## Role of Oncoproteomics in Biomarker Development

Oncoproteomics is the study of proteins and their interactions in a cancer cell by proteomic technologies [7, 8]. Two-dimensional gel electrophoresis is the first technique which has taken attention at the turn of the century; proteomics has been increasingly applied to cancer research with the wide-spread introduction of mass spectrometry and protein chip. Oncoproteomics involved in cancer is important for detection because of its specificity. Moreover, oncoproteomics is also applied to the discovery of new therapeutic targets and to the study of drug effects. The challenges ahead and perspectives of oncoproteomics for biomarkers development are also addressed with a wealth of information that can be applied to a broad spectrum of biomarker research projects. As an important biological indicator of cancer status and progression for the physiological state of the cell at a specific time, biomarkers represent powerful tools for monitoring the course of cancer and gauging the efficacy and safety of novel therapeutic agents. They may have tremendous therapeutic impact in clinical oncology, especially if the biomarker is detected before clinical symptoms or enables real-time monitoring of drug responses. Malignant transformation involves alterations in protein expression with subsequent clonal proliferation of the altered cells. These alterations can be monitored at the protein level, both qualitatively and quantitatively. Protein signatures in cancer provide valuable information that may be an aid to more effective diagnosis, prognosis, and therapy. The early detection of cancer has a potential to dramatically reduce mortality. The thermostable fractions of serum samples from patients with ovarian, uterus, and breast cancers, as well as samples from benign ovarian tumor were analyzed using two-dimensional gel electrophoresis (2-DE) combined with matrix-assisted laser desorption ionization-time of flight (MALDI-TOF) mass spectrometry. For example, alpha-1-acid glycoprotein and clusterin were expressly down-regulated in breast cancer, whereas transthyretin was decreased specifically in ovarian cancer. Polipoprotein A-I forms have decreased spot volume, while haptoglobin alpha1, in contrast, was elevated in several tumors. Serum thermostable fraction may be recommended as a good tool for medium and small protein investigation, in particular, by 2-DE [9]. The recent progress of proteomics has opened new avenues for cancer-related biomarker discovery. Advances in proteomics are contributing to the understanding of pathophysiology of neoplasia, cancer diagnosis, and anti cancer drug discovery. With the advent of new and improved proteomic technologies such as the development of quantitative proteomic methods, high-resolution, high-speed, high-throughput, high-sensitivity mass spectrometry (MS) and protein chip, as well as advanced bioinformatics for data handling and interpretation, it is possible to discover biomarkers that are able to reliably and accurately predict outcomes during cancer treatment and management [10]. Besides, these newer technologies provide higher analytical capabilities. Employing automated liquid handling systems, fractionation techniques and bioinformatics tools for greater sensitivity and resolving power, more robust and higher throughput sample processing, and greater confidence in analytical results can be obtained.

Oncoproteomics offers cutting-edge capabilities to accelerate the translation of basic discoveries into daily clinical practice. Continued refinement of techniques and methods to determine the abundance and status of proteins holds great promise for the future study of cancer and the development of cancer therapies. Sometimes it is difficult to identify the main cause of disease and it looks like needle in the haystack, on the other hand a magnet can localize the needle, so we can attach magnets to the molecules of interest and find out the needle. Recent studies have revealed that apart from the genetic abnormality of many cancer-related genes/proteins, epigenetic regulation of genes is also critically important in the process of carcinogenesis. The mechanisms of epigenetic control of genes involve changes of gene expression patterns mediated by modifications of DNA and/or histones, without the direct alteration of nucleotide sequence of the genes. Those modifications include several processes, mainly the DNA methylation and the covalent modifications such as acetylation, phosphorylation and ubiquitination of specific amino acid residues of the N-termini of the core histones. Among these modifications, histone acetylation/deacetylation plays a central role in epigenetic regulation of gene expression. Typically, high acetylation level of the chromatin hallmarks the active transcription of the genes, whereas transcriptionally inactive chromatin is usually characterized by low acetylation level of histones. It has been discovered that the occurrence of many cancers is accompanied by a genome-wide histone hypoacetylation and indeed, histone deacetylase inhibitors have significant and clinically proven anti-tumor activity [11].

## Currently Used Biomarker

Radioactive marker (radioactive elements like ^3^H, ^32^P, ^35^S, ^125^I), Optical marker (luminescence, fluorescence), Magnetic marker (magnet, nanoparticles, NMR) indirect electrical marker (conductivity) these are famous categories of currently used biomarkers in different fields. At the present stage various biomarkers are in use, some are classified in Table 2 [12]. Every category of biomarker has positive as well as negative characteristics, we can choose according to our need. When we deal with cancer early diagnosis is difficult because of the lack of specific symptoms in early stage of disease and the limited understanding of etiology and oncogenesis. For example, blood tumor markers for breast cancer such as cancer antigen 15-3 (CA15-3) are useless for early detection because of its low sensitivity. Therefore measurement of carcinoembryonic antigen (CEA) and HER-2 in abnormal nipple discharge has been approved for diagnosis of breast cancer in some countries. More than 98% of cervical cancer is related to human papillomavirus (HPV) infection. The identification and functional verification of host proteins associated with HPV E6 and E7 oncoproteins may provide useful information for the understanding of cervical carcinogenesis and the development of cervical cancer-specific markers. For hepatocellular carcinoma (HCC), the common methods of screening high risk patients by alpha-fetoprotein (AFP) and ultra-sonography has been shown to result in earlier detection and consequently more easily treatable tumors and longer survival. The newer high sensitive desgamma-carboxy-prothrombin has been found to be useful. In addition, the AFP fractions L3, P4/5, and the +II band are highly specific for HCC. Among routinely assayed tumor markers in the laboratory, CA-25 is more sensitive for HCC than AFP but far less specific. Currently available screening tests for ovarian cancer include CA-125, transvaginal ultrasound, or a combination of both. CA-125 has provided a useful serum tumor marker for monitoring response to chemotherapy. A rapid fall in CA-125 during chemotherapy predicts a favorable prognosis and can be used to redistribute patients on multi armed randomized clinical trials. Prostate-specific antigen (PSA) is the most important tumor marker in all solid tumors, indispensable in the management of prostate cancer [13]. However, most currently available screening tests for cancers lack high sensitivity and specificity to be useful in screening the general population, so the differentiation between some benign and malignant tumors is still a clinical challenge. The advent of oncoproteomics has provided the hope of discovering novel biomarkers for use in the screening, early diagnosis, and prediction of response to therapy.

**Table 2:** Currently Used Biomarkers in Different Fields

Types of cancer biomarker	Biomarker
1. Genetic biomarker	Biomarker of PTEN tumor suppressor gene status
	Gene mutation Oncogene
2. DNA biomarker	Gene amplification
	Microsatellite instability
	Mitochondrial DNA
	Viral DNA
3. RNA biomarkers: microRNAs	Protein biomarker
	B7 co-regulatory ligands
	Raised serum lactate dehydrogenase
	High-motility group protein A2
4. Metabolic biomarker	Hypoxia-induced factor-1
5. Epigenic biomarker: DNA methylation	Immunological biomarker: T-cell and cytokine responses
	Biomarker in cancer stem cells: crpto-1

## Newly Revealed Biomarker

Abnova and Japan National Cancer Center Research Institute have presented the result of the pancreatic cancer biomarker findings at the eighth Japan Human Proteome Organization (JHUPO) conference on July 26-27, 2010 in Tokyo, Japan [14]. This is the culmination of more than one and half years of research discovery and validation work utilizing antibody-based tissue microarray profiling to identify novel protein biomarkers which are preferentially expressed on cancer tissue followed by confirmation of the same proteins released into the blood stream. These biomarkers are benchmarked against the current standard biomarker, CA-19.9 for pancreatic cancer. The newly discovered biomarkers hold great value for early detection, diagnostic, prognostic, and theranostic applications in the management and treatment of pancreatic patients. Abnova has the exclusive right in the commercialization of the derived biomarkers and National Cancer Center Research Institute will receive royalty from sales of commercial products. Human tissue and sera are the two main sources of clinical sample available to clinicians and researchers for investigation of biomarkers which have gained increased importance in new era of personalized medicine. The approach undertook by Abnova and Japan National Cancer Center for the discovery of pancreatic cancer biomarkers is unprecedented. Abnova is a biotech company specializing in high throughput monoclonal antibody production. Japan National Cancer Center is a distinguished center of excellence for research and treatment of cancer patients. Leveraging Abnova’s large collection of monoclonal antibodies and National Cancer Center's well-maintained archive of human samples and clinical records, two parties joint force to apply more than one thousand monoclonal antibodies to pathological specimens and compared protein expression profiles of pancreatic cancer versus normal patients. Relevant protein biomarkers are then evaluated for their presence in sera by sandwich ELISA technique for quantitative analysis. Protein levels are also compared to the levels of normal individuals for evaluation and verification of statistical significance. For the most part, the antibody screening and tissue section staining can be automated. However, immunohistochemical interpretation and subsequent serum validation work have taken more time. The discovered biomarkers will challenge us on our understanding of pancreatic cancer and reinforce the complexity of biology and disease process. Such high throughput, antibody screening platform can be readily applied to all the major cancers for biomarker discovery and validation. It is an invaluable system for translational research and new diagnostic and therapeutic development. An interdisciplinary team of researchers from ETH Zurich, University Hospital Zurich and the Cantonal Hospital of St. Gallenhas has defined biomarkers in patients’ blood serum that indicates the presence of prostate cancer. The method used has the potential to be applied to other types of tumors [15]. A new European consortium called OncoTrack has just launched one of Europe's largest collaborative academic-industry research projects to develop and assess novel approaches for the identification of new biomarkers for colon cancer. The five year project, methods for systematic next generation oncology biomarker development, brings together top European academic researchers with a wide range of expertise and partners them with pharmaceutical companies [16].

If we talk about particular type of cancer like pancreas cancer it has the worst prognosis of any solid tumor but is potentially treatable if it is diagnosed at an early stage [17-18]. There are no population wide screening tests for pancreas cancer. The best established marker is CA 19-9 which is a sialylated Lewis antigen of the MUC1 protein with an overall sensitivity of 80% and specificity of 90%. Unfortunately, CA 19-9 may be positive in patients with non malignant diseases including cirrhosis, chronic pancreatitis, cholangitis, as well as other gastrointestinal cancers. Additionally, CA 19-9 is a poor screening test. At the Samsung Medical Center in South Korea 70 940 asymptomatic patients were screened using CA 19-9. However, among 1063 with elevated levels only 4 had pancreas cancer and only 2 had resectable disease. The Holy Grail for pancreas cancer investigators is to identify early markers which can predict the development of pancreas cancer, uncover early resectable disease, and guide therapy. As previously described CA 19-9 levels are inadequate to identify early pancreas cancer in the population.

## Future Aspect of Biomarker

In the age of technology future is bright for biomarkers. As various institutes are working on early detection of cancer there is possibility that we can detect cancer before the tumor formation. The scale of this attempt preclinical studies and large clinical trials is considerable, making it expensive. Collaboration with the pharmaceutical industries is essential because experimental anticancer drugs are an essential reagent for biomarker discovery experiments. Many cancer biomarkers will be broadly applicable (for example, they will not be restricted to predicting the response to a single drug), so a collaborative, precompetitive partnership with industry is warranted. Similar to government-sponsored projects such as the Human Genome Project and The Cancer Genome Atlas, early results from collaborative biomarker discovery projects should be released into the public domain to encourage further study and to avoid downstream intellectual property disputes that could delay commercialization efforts. It is time to establish an associative approach using a public-private partnership model to solve the cancer biomarker problem. All the stakeholders, patients, doctors, pharmaceutical and molecular diagnostic companies, regulatory agencies and health-care payers stand to benefit.

Universal cross sectional imaging is impractical and would be associated with high cost and potential radiation related morbidity. There are two major approaches to molecular marker discovery. In the high throughput “shotgun” strategies thousands of contenders are screened simultaneously. In the traditional hypothesis driven approach, interactions between molecules known to be important to pancreas cancer development are studied to identify novel molecules and pathways. When we talk about specific type of cancer prostate cancer (CaP) continues to be the second leading cause of cancer-specific death in men in Western countries. The marker currently used for CaP detection is an increase in serum prostate specific antigen (PSA). However, the PSA test may give false positive or negative information and does not allow the differentiation of benign prostate hyperplasia (BPH), non-aggressive CaP and aggressive CaP. Tears are a unique source of body fluid and contain proteins, peptides, mucins and lipids, which is useful for studying clinical proteomics.

Advances in the field of proteomics have greatly enhanced the study of tears, with a greater number of proteins now being identified in tears. Identification of novel biomarkers in tear is a new area of development. Modern advances in the field of proteomic techniques hold the promise of providing the clinical oncologists with new tools to find novel CaP biomarkers for early diagnosis and prognosis [19].

Early diagnosis of cancer needs focus on biomarkers identification. One possible approach may be the study of signaling system of pathways related with cancer in our body, as study of signaling system is not easy in itself; focus on one factor at a time is not an easy task. Relative study with respective to each factor that is at a time study of effect on one factor into another one and on whole process can be effective. Beside all these problems related with early diagnosis of cancer, biomarker identification is ultimate solution. We are in the age of technology and in future solution will be definitely found out. Many promising fields are growing day by day like nanotechnology and medical inventions, so we can hope for promising future ahead.
